# Synthesis of Indoleacetic Acid, Gibberellic Acid and ACC-Deaminase by *Mortierella* Strains Promote Winter Wheat Seedlings Growth under Different Conditions

**DOI:** 10.3390/ijms19103218

**Published:** 2018-10-18

**Authors:** Ewa Ozimek, Jolanta Jaroszuk-Ściseł, Justyna Bohacz, Teresa Korniłłowicz-Kowalska, Renata Tyśkiewicz, Anna Słomka, Artur Nowak, Agnieszka Hanaka

**Affiliations:** 1 Department of Environmental Microbiology, Institute of Microbiology and Biotechnology; Faculty of Biology and Biotechnology, Maria Curie-Sklodowska University, 20-033 Lublin, Poland; jolanta.jaroszuk-scisel@poczta.umcs.lublin.pl (J.J.-Ś.); renata.tyskiewicz@poczta.umcs.lublin.pl (R.T.); a.slomka@poczta.umcs.lublin.pl (A.S.); artur.nowak@poczta.umcs.lublin.pl (A.N.); 2 Department of Environmental Microbiology, Laboratory of Mycology, Faculty of Agrobioengineering, University of Life Sciences in Lublin, 20-069 Lublin, Poland; justyna.bohacz@up.lublin.pl (J.B.); teresa.kornilowicz@up.lublin.pl (T.K.-K.); 3 Department of Plant Physiology, Institute of Biology and Biochemistry, Faculty of Biology and Biotechnology, Maria Curie-Sklodowska University, 20-033 Lublin, Poland; agnieszka.hanaka@poczta.umcs.lublin.pl

**Keywords:** *Mortierella*, phytohormones, winter wheat seedlings, psychrotrophs

## Abstract

The endogenous pool of phytoregulators in plant tissues supplied with microbial secondary metabolites may be crucial for the development of winter wheat seedlings during cool springs. The phytohormones may be synthesized by psychrotrophic microorganisms in lower temperatures occurring in a temperate climate. Two fungal isolates from the Spitzbergen soils after the microscopic observations and “the internal transcribed spacer” (ITS) region molecular characterization were identified as *Mortierella* *antarctica* (MA DEM7) and *Mortierella verticillata* (MV DEM32). In order to study the synthesis of indoleacetic acid (IAA) and gibberellic acid (GA), *Mortierella* strains were grown on media supplemented with precursor of phytohormones tryptophan at 9, 15 °C, and 20 °C for nine days. The highest amount of IAA synthesis was identified in MV DEM32 nine-day-culture at 15 °C with 1.5 mM of tryptophan. At the same temperature (15 °C), the significant promoting effect (about 40% root and shoot fresh weight) of this strain on seedlings was observed. However, only MA DEM-7 had the ACC (1-aminocyclopropane-1-carboxylate) deaminase activity with the highest efficiency at 9 °C and synthesized IAA without tryptophan. Moreover, at the same conditions, the strain was confirmed to possess the strong promoting effect (about 40% root and 24% shoot fresh weight) on seedlings. Both strains synthesized GA in all tested terms and temperatures. The studied *Mortierella* strains had some important traits that led them to be considered as microbial biofertilizers components, improving plant growth in difficult temperate climates.

## 1. Introduction

In addition to *Penicillium* and *Aspergillus* genera*, Mortierella* are very common filamentous fungi isolated from the environment [1,2]. The studies on soil ecology describing fungal diversity reported an occurrence of *Mortierella* strains in soil, rhizosphere, rivers, and lakes on different continents [3,4,5,6,7,8,9]. Nagy et al. [1] described *Mortierella* as widespread in the temperate zone. Most of the strains investigated in detail were isolated from low-temperature environments. The temperature for psychrophilic microorganisms growth is about 5 °C or below, with the optimum at 15 °C or lower. In the case of psychrotrophs, they grow at a low temperature with the optimum one above 15 °C and maximal growth temperature above 20 °C [10,11].

Winter wheat is one of the most important cereal crops in Europe, mainly sown in fall. In Poland, the optimal seedling season for this plant is at the turn of the second half of September and the first half of October when the average daily temperatures correspond with the optimum growth temperature for psychrotrophs [12,13].

Indoleacetic acid (IAA) and gibberellic acid (GA), as well as ACC-deaminase, are plant growth regulators synthesized by plants as well as a number of soil microorganisms [14]. “IAA is essential for all important processes including seedlings growth. Tryptophan has been identified as a main precursor for IAA biosynthesis pathways in microorganisms (bacteria, fungi) and plants [15]. The dose of Trp recommended for fungal IAA synthesis is from 2 mM to 4 mM [16,17]. Many reports confirmed that even the very small amount of Trp may induce the IAA synthesis [15,18,19]. The term small dose is not defined and the lowest threshold value crucial for auxin synthesis could be strain-dependent.”

Among various functions, “GA” may stimulate the synthesis of hydrolytic enzymes activity during the germination of a cereal grain. Phytohormone ethylene plays role in plant development regulation and stress resistance. Moreover, the microbial ACC-deaminase converts the precursor of ethylene (ACC) to the ammonia and α-ketobutyrate and protects plants from excessive concentrations of ethylene [20,21,22].

The microbial production of phytohormones and phytoregulators, such as ACC-deaminase, is one of the most important direct mechanisms contributing to a rapid and durable soil colonization, which consequently enhances plant growth [23,24,25]. More attention should be paid to microorganisms promoting plant growth at lower temperatures, as well as in a broad range of temperatures during the selection of microbial components of biofertilizers to be applied in climate conditions of eastern Europe. There are a number of detailed studies on the synthesis of polyunsaturated fatty acids (PUFA) by fungi of the *Mortierella* genus*.* However, the literature data lack the information about the production of phytoregulators by *Mortierella* strains [26,27,28,29]. Therefore, in this study, we identified two *Mortierella* strains growing in a wide temperature range. We have also investigated the synthesis of IAA, GA, and ACC-deaminase and evaluated the effect of these microorganisms on the winter wheat seedlings growth in temperatures characteristic for the temperate zone.

## 2. Results

### 2.1. Identification and Optimal Growth Temperature of Fungal Isolates

**Figure 1:**
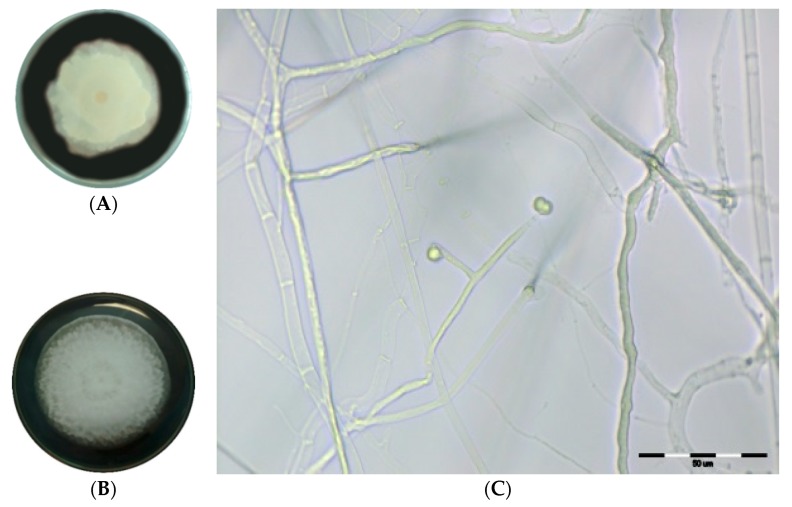
Mycelial growth of (**A**) *M. antarctica* DEM7; (**B**) *M. verticillata* DEM32 on potato dextrose agar (PDA) medium. (**C**) Sporangiophores with few-spored sporangia *M. verticillata* DEM32, “observed in microcultures on PDA medium, scale bar 50 µm”.

The mycelium of tested *Mortierella antarctica* (MA DEM7) and *Mortierella verticillata* (MV DEM32) fungi growing on potato dextrose agar (PDA) formed rosette, cottony, white aerial colonies (Figure 1A,B). Additionally, along with the MV DEM32 mycelium growth, the strain became yellowish. As a result of microscopic observations of both tested microcultures incubated at 20 °C for seven days, we did not obtain fungal sporulation. After seven days incubation at 4 °C, the MV DEM32 isolate formed characteristic sporangiophores with few-spored sporangia (Figure 1C). This strain (MV DEM32) was characterized by the presence of needle-shaped sporangiophores with two to three branches and few-spored sporangia.

In order to obtain the sporulation by starvation of *Mortierella* strains, the attempts were made to culture studied strains on the water agar. However, no growth was observed on this medium. Similarly, no good sporulation of MA DEM7 isolate was noticed.

The identification of fungal isolates the internal transcribed spacer (ITS) sequences was investigated and sent to NCBI GenBank. A similarity of 100% to *Mortierella antarctica* (strain MA DEM7) was detected. The MV DEM32 strain exhibition of 100% homology with *Mortierella verticillata* Linnem (the synonym for this species is *Mortierella marburgensis* Linnem) [30,31,32] was detected. According to the best of our knowledge, this is the first work describing *Mortierella verticillata* isolated from the Spitzbergen soil. The consensus of the ITS fragment (query) was submitted and deposited in the GenBank under the following accession numbers: MH 289781 (strain MA DEM7) and MH 304896 (strain MV DEM32).

### 2.2. The Effect of Temperature on the Radial Growth of Mortierella Strains

**Figure 2:**
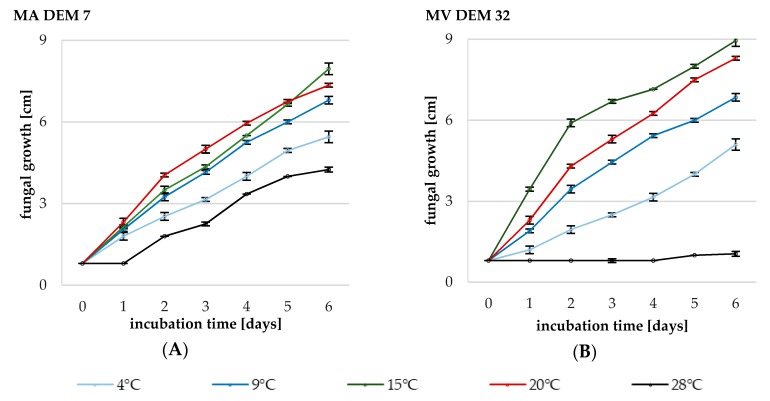
Mycelial growth [cm] at PDA medium measured daily for six days. (**A**) The diameter of the *M. antarctica* DEM7; (**B**) the diameter of the *M. verticillata* DEM32 at five temperature conditions. Values are means of three replicates. Bars represented standard deviations (SD).

The ability of two *Mortierella* strains to grow at five different temperatures was tested, and the optimal temperature conditions were determined (Figure 2A,B). The experiment was conducted for six days until the fungal colony “reached the maximum diameter of 9 cm of petri dish”. The results showed that MA DEM7 and MV DEM32 strains were able to grow at temperature range between 4 and 28 °C. However, the MA DEM32 demonstrated minimal growth at 28 °C (the diameter of colony on the sixth day of experiment was only 1 cm). The best temperature for mycelial growth of MA DEM7 and MV DEM32 was 15 °C, which led to the highest growth rates of 1.19 ± 0.08 and 1.35 ± 0.09, respectively (Table 1).

The *Mortierella* growth rate at 20 °C between the fifth and sixth day of the incubation was slow, as was indicated in our study. At 4 °C, the growth of the MA DEM7 and MV DEM32 colony was observed from the beginning of the experiment. The optimal growth temperature for *Mortierella* strains (MA DEM7 and MV DEM32) on PDA medium oscillated between 15 and 20 °C (Figure 2A,B).

### 2.3. Phytoregulators Activity Assayed at Different Temperature Conditions

**Figure 3:**
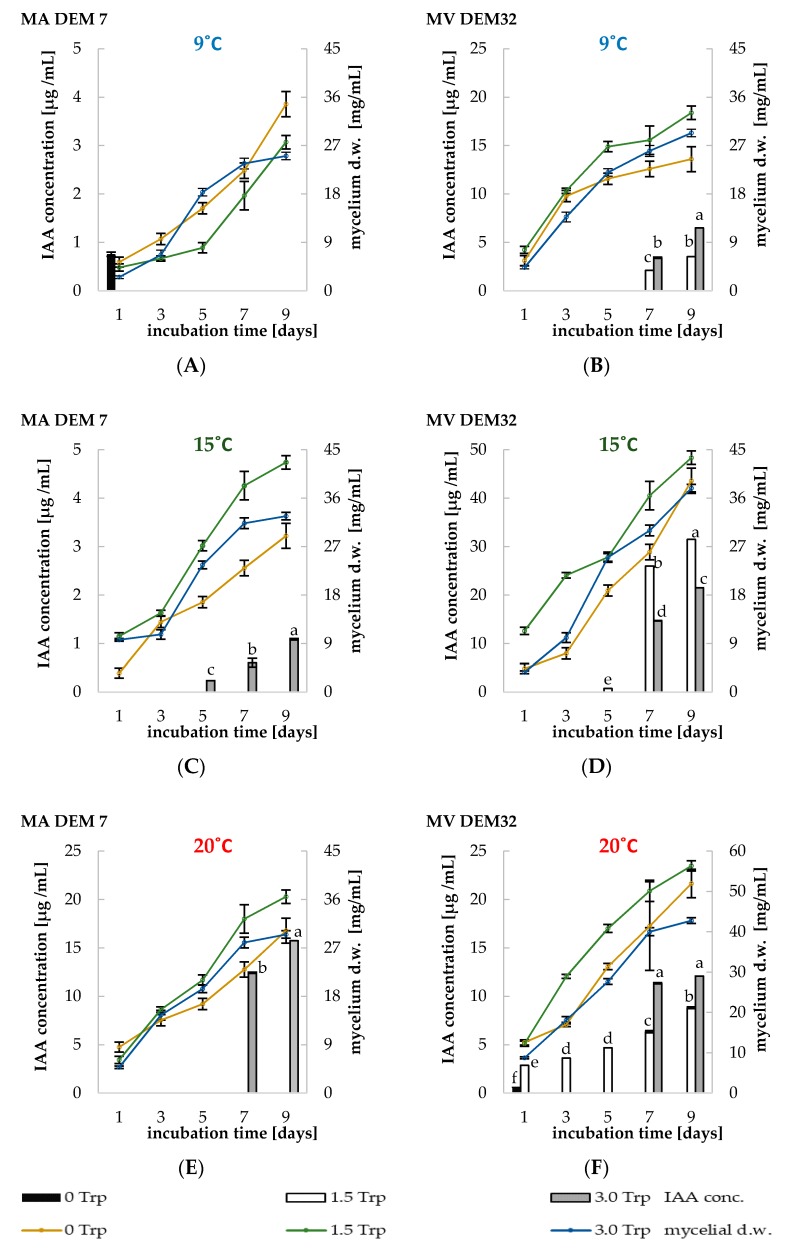
The indoleacetic acid (IAA) concentration and fungal biomas in *M. antarctica* DEM7 (**A**,**C**,**E**) and *M. verticillata* DEM32 (**B**,**D**,**F**) cultures grown in Chapek–Dox modified (CDM) medium supplemented with 1.5 and 3.0 mM, or without Trp in different temperature. Bars represented standard deviations (SD). Values followed by different letters are significantly different (one-way analysis of variance (ANOVA) followed by a Tukey’s post hoc test with the significance evaluated at *p <* 0.05).

Two fungal strains, MA DEM7 and MV DEM32, were tested for their ability to synthesize IAA. The CDM (Chapek–Dox modified) medium supplemented with an initial tryptophan (Trp) dose of 1.5 mM or 3.0 mM was more suitable for IAA production for MV DEM32 strain compared with CDM without Trp at “9 °C”, 15 °C, and 20 °C (Figure 3B,D,F). The highest amount of IAA was measured after the seventh and/or ninth day of *Mortierella* strains incubation at all temperature conditions (Figure 3B–F), except for the MA DEM7 strain incubated at 9 °C (Figure 3A). In the MV DEM32 cultures incubated at 9 and 20 °C, it was found that the concentration of IAA was correlated with the initial concentration of Trp (Figure 3B,F). The optimal growth conditions for both tested fungi were obtained in CDM supplemented with 1.5 mM Trp at all tested temperatures, except for the MA DEM7 strain incubated at 9 °C (Figure 3A). In the absence of Trp, only the *M. antarctica* strain synthetized (about 0.75 µg IAA/mL); however, the highest dry weight (about 38 mg/mL) of fungal biomass obtained at such conditions was observed. After the MA DEM7 strain incubation at 15 °C and 20 °C, the IAA synthesis was obtained, but only in cultures with the initial Trp dose of 3.0 mM (Figure 3C,E).

**Figure 4:**
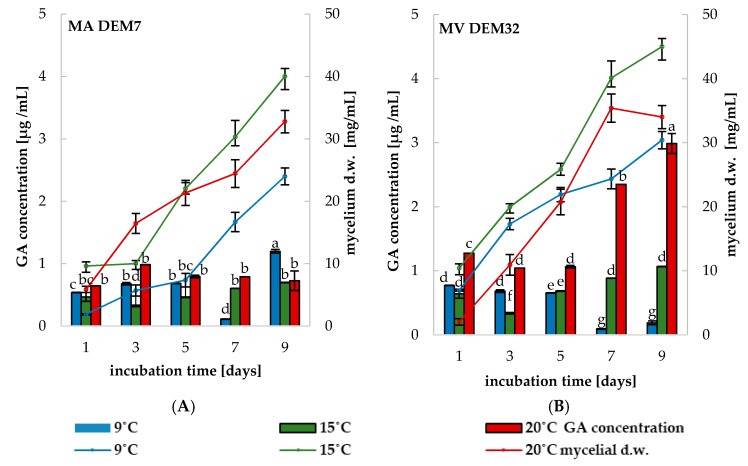
The gibberellic acid (GA) concentration and mycelial growth in *M. antarctica* DEM7 (**A**) and *M. verticillata* DEM32 (**B**) cultures grown in CDM medium supplemented with 3.0 mM Trp in different temperature conditions. Bars represented standard deviations (SD). Values followed by different letters are significantly different (one-way analysis of variance (ANOVA) followed by a Tukey’s post hoc test with the significance evaluated at *p <* 0.05).

Both *Mortierella* strains performed GA synthesis after 24 h of incubation on media supplemented with and without Trp in all three temperatures (9, 15 and 20 °C) (Figure 4A,B). The optimal conditions of phytohormone synthesis depended on the culture conditions. Under all tested conditions, the highest concentration of GA (about 3 µg/mL) was found after nine days of incubation at 20 °C for MV DEM32 isolate culture with 3.0 mM of Trp (Figure 4B). In the case of MA DEM7, the maximum concentration of GA was measured during the last day of incubation in the culture without prior addition of Trp (Figure 4A). The concentration of GA in MA DEM7 was three times lower (below 1 μg/mL) in a comparison with MV DEM32 at the same conditions (nine days, 20 °C). The optimal growth conditions for both tested *Mortierella* strains were similar (Figure 4A,B).

**Figure 5:**
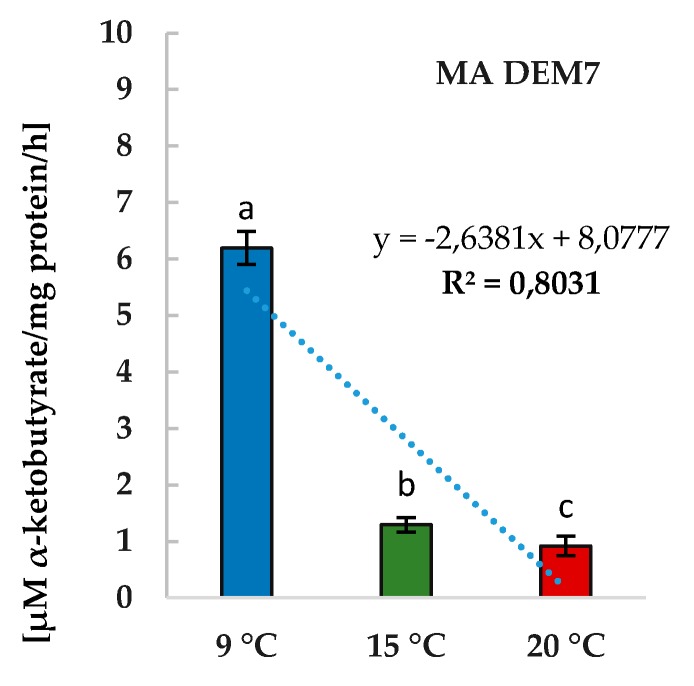
The ACC-deaminase activity of *M. antarctica* DEM7 culture in different temperature conditions (nine-day culture). Results are represented as µM of α-ketobutyrate mg/protein/h. Bars represented standard deviations (SD). Values followed by different letters are significantly different (one-way analysis of variance (ANOVA) followed by a Tukey’s post hoc test with the significance evaluated at *p <* 0.05).

Two *Mortierella* strains were able to grow on CDM (data not shown). Only *M. antarctica* DEM7 expressed ACC-deaminase activity from 0.9 to 6 µM of α-ketobutyrate/mg protein/h with the highest amount at 9 °C (Figure 5).

### 2.4. Growth of Winter Wheat Seedlings with Mortierella Strains under Various Conditions

**Figure 6:**
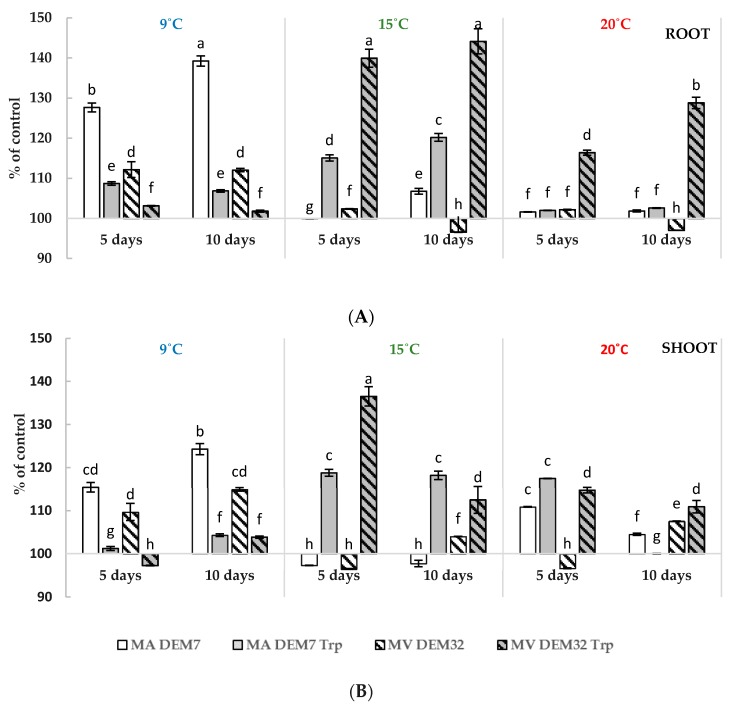
Effect of *Mortierella* strains on winter wheat seedlings root (**A**) and shoot (**B**) fresh weight in different temperature conditions with 3.0 mM Trp or without Trp. “The results of MA DEM7 and MV DEM32 are presented as a percent of control 1—not inoculated and not supplied with Trp; and results of MA DEM7 Trp and MV DEM32 Trp as a percent of control 2—not inoculated and supplied with Trp”. Bars represented standard deviations (SD). “Values followed by different letters are significantly different (one-way analysis of variance (ANOVA) followed by a Tukey’s post hoc test with the significance evaluated at *p <* 0.05).”

The winter wheat seeds in the presence of fungal inoculum with or without the initial dose of Trp were germinated in order to evaluate the ability of tested *Mortierella* strains to promote plant growth (Figure 6A,B). The cultivation of winter wheat grain at 15 °C with MA DEM32 strain in the presence of 3.0 mM Trp led to increase the total root fresh biomass to over 40% as compared with the control with Trp (Figure 6A). 

The same stimulating effect of MA DEM7 strain on root fresh weight was observed after 10 days of inoculation at 9 °C. Additionally, the shoot fresh weight of these plants was also higher without Trp (precursor of IAA)—as compared with the non-inoculated control and other plants growing without Trp (Figure 6B). The highest shoot fresh weight was measured in seedlings grown with the addition of Trp as well as MV DEM32 inoculum. After five days of development, the seedlings shoot fresh weight was almost 40% higher than the fresh weight of those grown aseptically with Trp.

**Figure 7:**
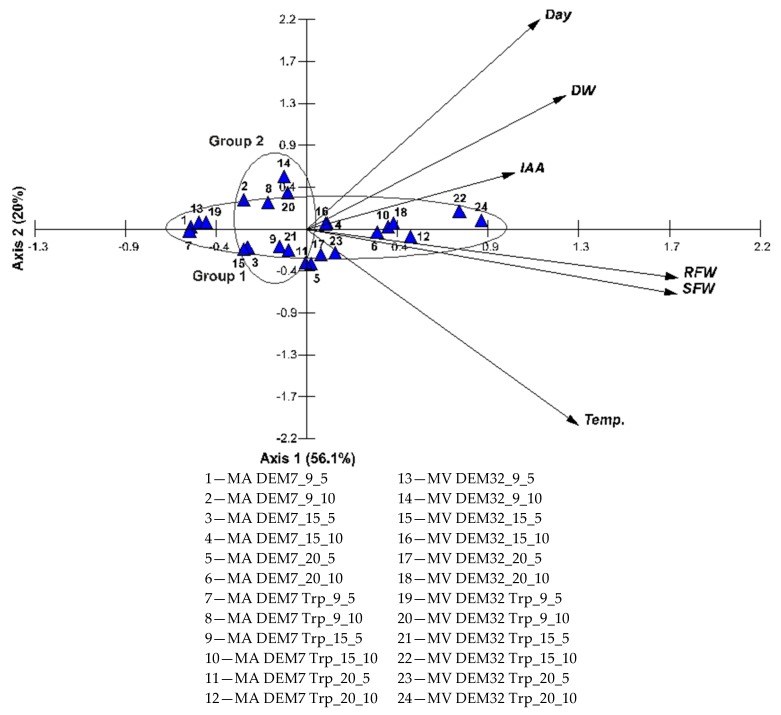
Ordination diagram showing the results of the principal component analysis (PCA) for the two strains: MA DEM7 and MV DEM23, both with out and with tryptophan, treated with three temperatures (9, 15, and 20 °C; Temp.), and analyzed in two days (5 and 10 day; Day). RFW and SFW—average root and shoot fresh weight, respectively; IAA—average concentration; SW—strain dry weight.

The principal component analysis (PCA) ordination analysis facilitated the determination of the relationships among IAA concentration, strain dry weight, and the roots and shoots fresh weight in two *Mortierella* strains, treated and not treated with tryptophan; cultivated at 9 °C, 15 °C, and 20 °C; and analyzed after 5 and 10 days (Figure 7). All the variables analyzed were statistically significant at the level of *p* < 0.05. Four axes of the diagram explained 98.1% of the data variability, that is, the first axis—56.1%, and the second one—20%.

The two main trends of the variation could be seen. The first trend was related to the first axis and was positively correlated with all variables tested. The strongest correlation with this axis was shown by average root fresh weight (RFW) and shoot fresh weight (SFW). Axis 1 determined the gradient of all analyzed parameters, “which was shown by the linear arrangement of the tested strains, defined as Group 1. The lowest values of RFW, SFW, DW (dry weight), and IAA were observed for 1 and 7, while the highest was observed for 22 and 24. The second axis showed the influence of variability of experimental conditions on the obtained results. “Temperature (Temp.) was strongly negatively correlated with the second axis, whereas Day was positively correlated with the discussed axis, thus both of these factors were in opposition to each other, which was shown in Group 2. Strain 14 was growing at 9 °C for 10 days, and strain 21 at 15 °C for 5 days, while strains 11 and 5 were growing at 20 °C for 5 days.” Group 1 represented an increasing dependence of FW starting from 7 and 1 to 22 and 24. Group 2 was negatively correlated with the first axis and correlated with the temperature. Axis 2 was strongly positively correlated with the day of the analysis. IAA concentration increased from 7 to 22 and 24.

## 3. Discussion

The good sporulation after seven days by incubating the microculture of MV DEM32 at 4 °C to trigger reproduction was obtained. According to Domsch et al. [30], sporangiophores of *M. verticillata* are smooth, more or less verticillately branched. Watanabe [32] noticed one to three sporangiospores per *M. verticillata* sporangium. An original isolate of *Mortierella verticillata* has a few two-spored sporangia [30]. The observation of MV DEM32 strain in our study confirmed the presence of needle-shaped sporangiophores, characterized by one to five branches, as was described by Skirgiełło et al. [31] (Figure 1C). According to phylogenetic analysis confirmed by DNA amplification and nucleotide sequence, Wagner et al. [33] classified *Mortierella antarctica* to group 6: alpina and polycephala. Domsch et al. [30] noticed that *Mortierella alpina* forms sporangium with a number of spores. Del Frate and Caretta [34] described the same features for *M. antarctica* and *M. alpina*. In the case of these strains, they form unbranched sporangiophores with a widened and often irregularly swollen base [30]. According to Del Frate and Caretta [34], *M. antarctica* additionally has a basal collarette. In our study, MA DEM7 failed to sporulate and some of the morphological characters of this species were not observed and, consequently, the genetic analysis was required. On the basis of ITS1 and ITS4 fragments analysis, two strains were identified as *Mortierella antarctica* (strain MA DEM7) and *Mortierella verticillata* (strain MV DEM32).

Five temperatures were applied to find the optimum one for mycelial growth of MA DEM7 and MV DEM32 (Figure 2A,B). The strains exhibited an optimal growth temperature between 15 °C and 20 °C. The results also confirmed that two *Mortierella* fungi are able to grow at lower temperatures (4 and 9 °C) and might be classified as psychrotrophs similarly to other isolates of *Mortierella* investigated in previous studies by Ali et al. [9] and Del Frate et al. [34]. Shmidt et al. [7] reported that 317-1 strain from subalpine forest, belonging to *Mortierellales*, has its optimal growth temperature between 15 °C and 20 °C*.* The strain of *Mortierella alpina* ITA-CCMA-952 isolated from Antarctic moss and tested by Melo et al. [4] grew at a very wide range of temperatures with the optimum between 15 °C and 25 °C. The strain incubated for six days at 4 °C reached about 2.8 cm in diameter; however, no grow of the colony of this Antarctic fungus was observed for the first two days of the experiment. After six days at 15 °C, the *M. alpina* ITA-CCMA-952 colonies reached only 4.0 cm, which was only about 50% of final MA DEM7 and MV DEM32 diameter (Figure 2A,B). These data showed that the optimal growth temperature of strains isolated from similar habitat may differ significantly.

There are a number of reports indicating that plant growth promoting fungi (PGPF) improve crop productivity [4,25,35,36,37,38]. The IAA, the main auxin, is crucial for the majority of plant growth and development processes “and it is also a major factor determining the competition between fungal species that occupy the same niche [15]”. The production of microbial IAA may depend on the presence of its precursor, tryptophan; however, some microorganisms and plants can also synthesize IAA by Trp-independent mechanism [20,39]. According to Quiroz-Villareal et al. [16], this amino acid was identified as one of the components of root exudates. The IAA synthesis is widespread among soil microorganisms involved in a plant growth promotion. It was found that the concentration of IAA in “MV DEM32” cultures was correlated with the initial dose of Trp at “9 °C” and 20 °C “(Figure 3B,F), but in the presence of 1.5 mM of Trp, the MV DEM32 strain synthesized the highest amount of phytohormone (about 32 µg mL) (Figure 3D). Many reports confirmed that even the very small amount of Trp may induce the IAA synthesis, but in fact, the term ‘small dose’ is not clearly defined [15,17,20,39]. Kumar et al. [18] announced the IAA concentrations in cultures of bacteria with the 4 mM dose of Trp, whereas Khan et al. [39] applied 0.4 mM of IAA precursor.” The optimal dose of Trp recommended for fungal cultures is from 2 mM to 4 mM and the maximal concentration of IAA was usually measured after 60 and 72 h of incubation [17,40], “whereas the lowest threshold value indispensable for auxin synthesis is not defined. The MA DEM7 strain did not synthesize the IAA on the CDM media with 1.5 mM of Trp, even though the mycelial growth was observed in all three temperatures (Figure 3A,C,E). In the presence of 3 mM of Trp, MA DEM7 culture synthesized even 16 µg/mL IAA (Figure 3E). In our previous studies concerning the IAA synthesis by mycelial fungi, the initial doses of Trp in the cultures were 0.3 mM, 6 mM, or 9 mM. It has been demonstrated that the optimal conditions for the IAA synthesis depended on the dose of Trp, time of incubation, and *Fusarium culmorum* strain [20]”. The latest publication indicated the significant increase of IAA levels in a tissue of maize seedlings growing in soil inoculated with *Mortierella elongata* [41]. The concentration of IAA in a plant tissue inoculated with this strain after 29 days was about 27% higher than that measured in non-inoculated seedlings. Furthermore, the whole genome sequencing of *Mortierella elongata* showed the genetic capacity to synthesize the IAA. Wani et al. [37] investigated the IAA synthesis of fungal isolates from *Croccus sativus* in India. On the seventh day of incubation, the *Mortierella alpina* CS10E endophytic strain synthesized IAA in the amount of 21.6 mg/L, while our *M. verticillata* DEM32 strains produced about 26 mg/L at the same time (Figure 3D).

GA (diterpenoid acid) is one of the major phytoregulators, among others, seed germination control, as well as root and shoot growth of plants [42]. GA has an influence on the plant growth at very low concentrations and is known to be synthesized by plants and some microorganisms. There are many reports indicating the ability of filamentous fungi belonging to genera: *Aspergillus, Penicillium, Fusarium*, and *Rhizopus* to synthesize GA acid [20,42,43,44]. According to the best of our knowledge, this is one of the first works investigating the synthesis of GA by *Mortierella* strains. The concentration of GA indicated in MV DEM32 cultures was correlated with the temperature of incubation (Figure 4B). Moreover, the highest value of phytohormone measured at 20 °C was about three times higher in a comparison with the amounts of GA in the cultures incubated at 9 °C “(about 0.8 µg/mL)” or/and 15 °C “(about 1.0 µg/mL)”. The MV DEM32 synthesis of GA at 20 °C was greater every day in a contrast to the highest amount of GA measured on the first day of incubation. It is important to take into account an individual time of phytoregulators accumulation in liquid cultures when selecting PGPF strains. Additionally, the dry weight of MV DEM32 mycelium measured on ninth day cultures incubated at 9 °C and 20 °C was about 37 mg/mL. These results showed that the synthesis of GA depends on the temperature. Fluctuations in the results of phytohormone synthesis observed in culture of MA DEM7 strain in all temperatures (Figure 4A) suggested that some part of GA acid could be utilized [37]. Our previous studies with a filamentous fungi suggested that Trp (the precursor of IAA and GA) may be consumed [20].

ACC-deaminase is one of efficient markers for microorganisms, which promote plant growth by decreasing the level of ethylene in plant tissues [45]. One of the products of ACC hydrolysis is ammonia, the source of nitrogen available for microorganisms and plants. The positive effect of inoculation with rhizobacteria containing ACC-deaminase was observed [14]. In the presented study, MA DEM7 strain increased the fresh biomass of seedlings root at 9 °C (Figure 5A). The linear correlation between the temperature of incubation and ACC-deaminase activity (*R*^2^ = 0.803) implied that the activity of phytoregulator increased with the decrease of the incubation temperature.

The production of phytoregulators may enhance the germination, as well as root and shoot plant growth [43]. Our results of IAA activity by MA DEM7 and MV DEM32 suggested that both strains could effectively promote plant growth at lower temperature. In order to study the real *Mortierella* impact on the growth of winter wheat seedlings, seeds with strains in the presence of Trp were inoculated. Plant roots are very sensitive to IAA concentration and they are the first to respond to the increase of exogenous auxin amount [46]. In this study, an inoculation with studied *Mortierella* fungi at 15 °C and 20 °C with 3.0 mM Trp, and after five days, the increase in fresh weight of root was shown and this effect was also visible after 10 days (Figure 6A,B) as compared with not inoculated seedlings.

In an experiment performed by Ul Hassan and Bano [37], the seeds of *Triticum aestivum* were treated with aqueous solution of Trp. After 57 days of pot experiments, the weight of fresh aerial parts of plants treated with *Pseudomonas moraviensis* or *Bacillus cereus* increased by about 20% as compared with the control. In this study, inoculation with *Mortierella* strains improved shoot growth of winter wheat seedlings (*Triticum aestivum* L. cv Arkadia) under various temperature conditions (Figure 6B). Additionally, higher root and shoot biomass of plants mainly cultivated at 15 °C and 20 °C with initial dose of Trp were noticed, whereas the highest fresh mass of plants cultivated without Trp was measured at 9 °C. These data suggest that at lower temperatures, the presence of the precursor of phytohormones is not the main factor influencing the phytoregulators synthesis.

Winter grains germinate until the beginning of winter and the plant root is crucial for good plant adaptations to critical factors characteristic for the temperate zone. Some of psychrotolerant *Mortierella* strains may enhance the plant growth at the beginning of a vegetative period in the temperate zone. It is concluded that further studies using these strains should be continued for the exact contribution of the other PGP traits of *Mortierella* strains at lower temperatures. The potential of these strains in a practical use (in field experiment) should be undertaken. According to our research, the *M. verticillata* DEM32 strain indicated phytohormones synthesis and stimulated growth of plant biomass especially at 15 and 20 °C in the presence of Trp (Figure 4A,B). What is interesting is that *M. antarctica* DEM7 showed its highest ACC-deaminase activity and GA synthesis at 9 °C in a comparison with all temperatures studied. The selection of microbial components of biofertilizers should be based on the wide spectrum of biological diversity [47]. On this account, the PGPF activity in lower temperatures should be also taken into consideration. There is still a need to perform studies on efficient strains potential component of an agricultural product.

## 4. Materials and Methods

### 4.1. Microscopic and Molecular Identification of Fungal Isolates

The experiment was carried out on two *Mortierella* fungi MA DEM7 and MV DEM32 isolated from soil humus horizon collected from micro-relief forms in tundra in the Bellsund region, west coast of Spitzbergen, in the Svalbard Archipelago (77°33′ N, 14°31′ E). Soil properties and microbial activity were presented and discussed in the study by Kurek et al. [10].

The morphological identification of pure cultures of isolates to the species level was accomplished using established procedures including macroscopic characterization on the plates and microscopic observation in the microcultures. In the macroscopic observations of cultures on plates and slants on PDA medium, the following macromorphological traits were considered for colonies size and structure: velvety, woolly, or the presence of concentric petals or stipes; pigment (revers, awers). The micromorphological features included the observation of the shape and size of sporangiophores, sporangia, and the amount of sporangiospores in sporangium, as well as the presence and morphology of chlamydospores. Fungal microcultures were cultivated in sterile petri dishes with a sterile triangle-shaped glass rod and microscope slide. Agar discs (10 mm in diameter) were cut with sterile cork-borer from PDA medium and placed on the center of the slide. Small blocks of medium were inoculated with mycelial hyphe of the *Mortierella* fungi. After that, sterile cover slide was put onto inoculum. The sterile distilled water (3 mL) was transferred gently on the bottom of the petri dish in order to maintain humidity. Microcultures were incubated at 20 °C for seven days, as well as at 4 °C for seven days. To obtain a better sporulation of tested fungi, attempts were made to culture microorganisms on water agar (agar media containing no nutrients) and incubate at 4 °C and 20 °C, according to Su et al. [48].

The morphological characteristics and classification to the species level were determined according to the keys and descriptions provided by Domsch et al. [30], Watanabe [32], and Skirgiełło et al. [26]. The microscopic observations were conducted in microcultures on agar discs using an Olympus BX-41 laboratory microscope [49].

### 4.2. Genomic DNA Extraction, Primers, and Sequencing

Fungal species were identified using the ITS (internal transcribed spacer) region sequencing method. The total genomic DNA was extracted from fungal mycelium using CHELEX resin (Biorad, Hercules, CA, USA) and enzymes for digesting the cell wall, that is, lyticase (1 mg/mL) and Proteinase K (20 mg/mL). The ITS region was amplified using two primers described by White et al. [50]. The ITS1 (59′-TCC GTA GGT GAA CCT GCG G-39′) and ITS 4 (59′-TCC TCC GCT TAT TGA TAT G-39′) make use of conserved regions of the 18S (ITS 1) and the 28S (ITS 4) rRNA genes to amplify the intervening 5.8S gene and the ITS 1 and ITS 2 noncoding regions. The primers were synthesized using the UNMC, Eppley Molecular Biology Core Laboratory. The amplicons were resolved by a capillary electrophoresis using the Applied Biosystems Inc. (Foster City, CA, USA). ABI3730xl DNA genetic analyser in the DNA Sequencing and Oligonucleotide Synthesis Laboratory, Institute of Biochemistry and Biophysics oligo.pl in Polish Academy of Sciences, Warsaw, Poland. Contiguous sequences (contigs) were assembled from chromatogram sequence data using Seqman (DNAStar, Madison, WI, USA), and a consensus sequence was generated. The sequences were aligned with a software aligner and analyzed in order to identify the fungi according to BLAST search of the sequences and compared with the NCBI GenBank database. The nucleotide sequences are available under the following GenBank accession numbers: MH 289781 (strain MA DEM7); MH 304896 (strain MV DEM32).

### 4.3. Determination of Optimal Fungal Growth Temperature

The initial cultures of *M. antarctica* DEM7 and *M. verticillata* DEM32 were incubated at 4, 9, 15, 20 and 28 °C on PDA medium for six days. To determinate the optimal growth temperature, the *Mortierella* isolates were grown on PDA medium (potato dextrose agar, Sigma-Aldrich, St. Louis, MI, USA) at five temperatures (4, 9, 15, 20, and 28 °C). The mycelia discs (0.8 cm) from initial cultures were transferred to PDA medium in petri dishes (total diameter of 9 cm). The diameters of the colonies of *Mortierella* strains were measured daily for six days. Data obtained for mycelial growth were collected from six replicates.

### 4.4. Maintenance of Strains

The *Mortierella* strains were stored on PDA (potato dextrose agar, Sigma-Aldrich, St. Louis, MI, USA) slants at 4 °C; transferred every three months; and deposited in the Fungal Collection of the Department of Environmental Microbiology (DEM) at Maria Curie-Sklodowska University, Lublin, Poland.

### 4.5. In Vitro Screening of Mortierella Strains for Phytoregulators Synthesis

#### 4.5.1. Preparation of Fungal Inoculum

In order to prepare a liquid inoculum, two *Mortierella* strains (MA DEM7 and MV DEM32) were grown on CDM (Czapek–Dox modified) medium composed of the following: glucose, 10.0 g; NaNO_3_, 3.00 g; K_2_HPO_4_, 1.00 g; MgSO_4_, 0.50 g; KCl, 0.50 g; and FeSO_4_, 0.01 g in 1000 mL, pH 7.0 [51]. The strains were cultivated in darkness at 20 °C and 60% relative humidity in an Innova 4900 growth chamber (New Brunswick Scientific, Edison, NJ, USA) at 120 rpm for three days. Mycelial pellets from shaken cultures were filtered from the growth medium and then washed and re-suspended in 9 mL sterile, deionized water. The mycelial suspensions were fragmented under sterile conditions five times for 20 s at 1-min intervals at 10,000 rpm in a laboratory blender (IKA^®^ T 18 basic ULTRA—TURRAX^®^), and diluted to the desired concentration (about 1 × 10^5^ cfu/mL). To confirm the number of fungal cfu/mL, 1 mL of the homogenate of the biomass was suspended in 9 mL of sterile distilled water and mixed vigorously with a vortex, and the serial dilutions were prepared. The number of fungal cfu/mL was determined by the plate dilution technique on CDM medium and counted after seven days of incubation at 20 °C.

#### 4.5.2. The Phytohormones and ACC-Deaminase Formation

To study the formation, the IAA and GA *Mortierella* strains were cultivated in 250 mL Erlenmeyer flasks with 50 mL of a CDM liquid media (pH 7.0). The CDM medium was supplemented with the solutions of IAA precursor—Trp (0 mM, 1.5 mM, and 3.0 mM) sterilized using syringe filters (0.22 μm). Cultures were incubated at 9 °C, 15 °C, and 20 °C. To study the GA synthesis, strains were cultivated on CDM medium with initial dose of 3.0 mM Trp. The ACC-deaminase activity was studied on CDM medium with 1.5 g of NaNO_3_. The phytohormones concentration were measured in “fungal culture supernatants” and ACC-deaminase activity were measured in “homogenized fungal culture suspensions” after 1, 3, 5, 7, and 9 days. The dry weight of mycelial fragments in the liquid culture was determined after collecting on Whatman no. 1 filters and weighting after drying at 60 °C for 24 h. To determine the ability of the *Mortierella* strains to synthesize IAA, GA, and ACC-deaminase, the supernatants of the liquid culture were centrifuged (10,000*×* *g* for 10 min). The IAA concentration was estimated according to the method of Glickmann and Dessaux [52], using Salkowski’s modified reagent [20,53]. 1mL of the culture supernatants was mixed with 1 mL of Salkowski’s reagent. The absorbance of the pink color was developed after 30 min of incubation in darkness at 20 °C at *λ* = 530 nm. The concentration of IAA was calculated from the regression equation of a standard curve of pure indole-3-acetic acid (Sigma-Aldrich, St. Louis, MI, USA. To quantify the GA using the modified method of Brückner et al. [35,54] and Hasan [44], the pH of filtrates was adjusted to 2.8 with 1 M HCl and extracted three times with equal volumes of ethyl acetate. The ethyl acetate fractions were collected and vacuum-evaporated (using Eppendorf Concentrator plus) at 20 °C. The dry pellet was re-suspended in ethanol/H_2_SO_4_ (9:1, *v/v*) mixture. The absorbance was measured at *λ* = 254 nm and the GA concentration was calculated from the regression equation of a standard curve of pure gibberellic acid (Sigma-Aldrich, St. Louis, MI, USA) and expressed as µg/mL [20]. All the spectrophotometric analyses were conducted on Varian Cary 1E UV-Visible Spectrophotometer.

The ACC-deaminase activity was assayed according to a modified method of Belimov et al. [55] and Shaharoona et al. [14], measuring the amount of α-ketobutyrate product released after hydrolysis of ACC. The number of μmol of α-ketobutyrate produced during this reaction was determined by comparing the absorbance at 540 nm of a sample to a standard curve of α-ketobutyrate (Sigma-Aldrich, St. Louis, MI, USA) ranging between 0.1 μM to 1.0 µM. The ACC-deaminase activity was expressed in mM of a-ketobutyrate mg/protein/h. The protein concentration was quantified by the Bradford method [56]. For the assessment of ACC-deaminase activity, *Mortierella* strains were grown on CDM medium at 9 °C, 15 °C, and 20 °C. At the end of the induction period, the cultures were centrifugated at 15,000 rpm for 5 min, and washed with 0.1 M Tris-HCl (pH 7.5). After centrifugation, the mycelia were fragmented under sterile conditions, three times for 10 s at 1-min intervals at 10,000 rpm in a laboratory blender (IKA^®^ T 18 basic ULTRA—TURRAX^®^). Then, 25 µL of toluene was added to 200 µL of homogenized material and vortexed vigorously for 30 s. ACC (20 µL of 0.5 M solution) was added to the suspension; vortexed; and after 15 min incubation at 20 °C, 1 mL of 0.56 N HCl was mixed. The lysates were centrifuged (10,000 *g*, 10 min) and 1 mL of the supernatant was mixed with 800 mL of 0.56 N HCl and 300 mL of 2,4-dinitrophenylhydrazine (0.2 g in 100 mL of 2 N HCl). The mixtures were incubated for 30 min at 20 °C, followed by the addition of 2 mL of 2 N NaOH and mixing. The absorbance of the mixture was measured using spectrophotometer at 540 nm.

### 4.6. Winter Wheat Seedlings Inoculation with Mortierella Strains—Experimental Design

Seeds of winter wheat (*Triticum aestivum* L. cv Arkadia) purchased from DANKO Plant breeding Choryń Poland) were used in the experiments. Grains were surface-sterilized according to the procedure described by Jaroszuk-Ściseł and Kurek [57]. For 30 min, the seeds were washed in tap water, then soaked for 10 min in a solution of HgCl_2_ (0.1%, *w*/*v*), submerged for 10 min in a solution of H_2_O_2_ (30%, *v/v*), and washed three times with sterile deionized water. Surface-sterilized seeds were rinsed ten times in homogenized mycelial inoculum of MA DEM7 and MV DEM32 (about 1 × 10^5^ cfu/mL), prepared as described before (Section 4.5.1), and transferred to sterile petri dishes taking 20 seeds per dish. Six experiment treatments were used: “control (1)—not inoculated and not supplemented with Trp; control (2)—not inoculated and supplemented with Trp; (3) MA DEM7 Trp—inoculated with MA DEM7 and supplemented with Trp; (4) MV DEM32 Trp—inoculated with MV DEM32 and supplemented with Trp; (5) MA DEM7—inoculated with MA DEM7 and not supplemented with Trp; and (6) MV DEM32—inoculated with MV DEM32 and not supplemented with Trp”. The effect of fungal strains on winter wheat seedlings growth was observed under different temperature conditions (incubation at 9, 15, and 20 °C). The root and shoot fresh total weight of winter wheat seedlings was measured in the unit of g after 5 and 10 days of incubation. “This experiment was carried out in three independent repeats with six replicates of each treatment.”

### 4.7. Statistical Analysis

The experiments were carried out in three independent replicates and the results were expressed as mean ± SD. Standard deviations (presented as deviation bars) were determined using Microsoft Excel 2016 (Microsoft Corp., Redmond, Washington, DC, USA). “The data were subjected to one-way analysis of variance (ANOVA) followed by a Tukey’s post hoc test where applicable, with the significance evaluated at *p <* 0.05.” The responses of *Mortierella* strains to the selected temperatures and cultivation days were evaluated by the principal component analysis (PCA) in the MVSP3.21 package. The analyses were based on the average values.

## 5. Conclusions

On the basis of morphological properties and sequence homology of the MA DEM7 and MV DEM32 filamentous fungi, two isolates from Spitzbergen soil were identified as *Mortierella antarctica* and *Mortierella verticillata*, respectively. The results obtained in our study confirmed the ability of studied *Mortierella* strains to grow and synthetize the phytoregulators in conditions characteristic for a temperate climate**.** After the winter wheat inoculation with *Mortierella* strains, a positive effect on seedlings root and shoot fresh mass was observed.

## Figures and Tables

**Table 1:** The mycelial growth rate [cm/day] of *M. antarctica* DEM7 and *M verticillata* DEM32 at five temperature conditions.

Temperature	MA DEM7	MV DEM32
4 °C	0.77 ± 0.06 ^c^	0.71 ± 0.02 ^c^
9 °C	1.0 ± 0.05 ^b^	1.0 ± 0.04 ^b^
15 °C	1.19 ± 0.08 ^a^	1.35 ± 0.09 ^a^
20 °C	1.09 ± 0.05 ^ab^	1.25 ± 0.06 ^ab^
28 °C	0.7 ± 0.02 ^d^	0.041 ± 0.00 ^d^

Values followed by different letters are significantly different (one-way analysis of variance (ANOVA) followed by a Tukey’s post hoc test with the significance evaluated at *p <* 0.05).

## Abbreviations

IAA	indoleacetic acid
GA	gibberellic acid
ACC deaminase	1-aminocyclopropane-1-carboxylate deaminase
ITS	internal transcribed spacer
PGPF	plant growth promoting fungi
PDA	potato dextrose agar
CDM	Chapek-Dox modified medium
